# Automated Quantitative Immunofluorescence Microscopy Approach for Diagnosis of Hereditary Thrombopathies: A Proof of Concept Using Bernard–Soulier Syndrome and Glanzmann Thrombasthenia

**DOI:** 10.3390/genes16060621

**Published:** 2025-05-23

**Authors:** Kevin Loos, Rawya Al-Rifai, Sandra Ohlenforst, Claudia Klein, Johannes Oldenburg, Anna Pavlova, Behnaz Pezeshkpoor

**Affiliations:** Institute of Experimental Hematology and Transfusion Medicine, University Clinic Bonn, 53127 Bonn, Germany; s4keloos@uni-bonn.de (K.L.); rawya.al_rifai@ukbonn.de (R.A.-R.); sandra.ohlenforst@ukbonn.de (S.O.); claudia.klein@ukbonn.de (C.K.); johannes.oldenburg@ukbonn.de (J.O.); anna.pavlova@ukbonn.de (A.P.)

**Keywords:** inherited platelet disorders, Bernard–Soulier syndrome, Glanzmann thrombasthenia, immunofluorescence microscopy, automated diagnosis, flow cytometry, thrombocytopathy

## Abstract

Inherited platelet disorders (IPDs) are rare bleeding disorders characterized by impaired platelet function and/or reduced blood platelet count. Their diagnosis typically relies on complex laboratory methods, including flow cytometry, aggregometry, and molecular genetic analysis. In recent years, immunofluorescence microscopy has been established as an alternative diagnostic method for IPDs. **Background/Objectives**: This study aims to validate a quantitative approach enhancing reproducibility through automated image analysis for diagnosing IPDs using immunofluorescence microscopy, with Bernard–Soulier Syndrome (BSS) and Glanzmann thrombasthenia (GT) as model IPDs. **Methods**: Native blood smears from patients with suspected BSS or GT were stained using a standardized immunofluorescence protocol targeting platelet surface glycoproteins, granules, and cytoskeletal components. The slides were analyzed using an automated fluorescence microscope, and a rule-based subpopulation analysis was implemented to quantify fluorescence signals. The results were compared to those of a healthy control group, as well as data from flow cytometry and molecular genetic testing. **Results**: The automated analysis successfully differentiated BSS and GT patients from healthy controls based on distinct fluorescence signal patterns. In BSS samples, CD42b (GPIbα) expression was absent or severely reduced, while GT samples showed a deficiency of CD41/CD61 (GPIIb/IIIa). The platelet size distribution confirmed macrothrombocytopenia in BSS patients. Flow cytometry and molecular genetic testing corroborated these findings, supporting the diagnostic reliability of the automated immunofluorescence microscopy approach. **Conclusions**: This proof-of-principle study demonstrates that automated quantitative immunofluorescence microscopy is a viable alternative for diagnosing IPDs, offering a standardized, objective, and efficient method, particularly in settings where flow cytometry is not feasible.

## 1. Introduction

Inherited platelet disorders (IPDs) encompass a rare and heterogeneous group of clinical manifestations characterized by quantitative and/or qualitative platelet defects, often resulting in variable bleeding tendencies [1,2,3]. Moreover, platelets play a central role in thrombosis, and elevated levels of reticulated platelets, which are newly released from the bone marrow, have been associated with increased platelet turnover, insufficient response to antiplatelet therapy, and a higher risk of adverse cardiovascular events [4,5]. Atherosclerosis is also strongly dependent on platelet activation and aggregation for its development and progression [5,6,7,8].

Thrombocytopenia is easily detected by routine cell analyzers, while platelet function disorders require advanced tests [9]. The current guidelines for the diagnosis of platelet function disorders recommend a stepwise diagnostic approach, starting with anamnesis, followed by coagulation tests, platelet count determination, mean platelet volume (MPV), immature platelet fraction (IPF), and platelet size distribution analysis [10]. A May–Grünwald–Giemsa-stained blood smear serves as the first morphological assessment, and specialized techniques such as aggregometry, flow cytometry, molecular genetics, and immunofluorescence (IF) microscopy represent the final steps in the diagnostic algorithm [11,12]. However, these specialized tests require fresh samples, the availability of infrastructure, and skilled personnel, which can limit accessibility, particularly in under-resourced or remote settings.

Bernard–Soulier syndrome (BSS) [13] and Glanzmann thrombasthenia (GT) [14] are two well-characterized platelet disorders distinguished by specific deficiencies in platelet membrane glycoproteins. Both IPDs are rare, autosomal recessive inherited disorders classified as thrombocytopathies, with an estimated incidence of approximately 1 in a million, increasing up to 1 in 200,000 in populations with high rates of consanguinity [2,13,15].

BSS is characterized by giant platelets with a combination of impaired platelet function and thrombocytopenia [13,16]. It is further subdivided based on the expression of the glycoprotein Ib–IX–V complex. While heterozygous carriers remain clinically asymptomatic, homozygous individuals present with a bleeding tendency from early childhood [17,18]. Since the discovery of the first mutation [19], over 203 pathogenic alterations have been identified in *GP1BA* (91), *GP1BB* (72), and *GP9* (40) genes, encoding for the GPIbα, GPIbb, and GPIX subunits, respectively [16,17]. Most of the mutations prevent the coordinated association of the complex, resulting in a very low expression of GPIb/IX/V itself on the platelet membrane [20,21].

GT is an autosomal recessive disorder of the platelet surface receptor GPIIb/IIIa (αIIbβ3), expressed either as a qualitative or quantitative defect, which results in defective platelet aggregation and diminished clot retraction [14,22]. These integrins are encoded by the *ITGA2B* and *ITGB3* genes and together form the platelet glycoprotein (GP)IIb/IIIa, which acts as the principal platelet receptor for fibrinogen [23]. Type I is the most common subtype, accounting for around 78% of patients with GT type II and type III (functional variant in receptor), constituting around 14% and 8% of cases, respectively [24].

Although the guidelines still recommend flow cytometry analysis as a first-line diagnostic approach for these two most common IPDs, in recent decades, IF microscopy has emerged as a powerful adjunct, or even an alternative, diagnostic method, particularly when access to flow cytometry is limited [25,26]. The key advantage of IF microscopy over flow cytometry and aggregometry is its lower pre-analytical restrictions, as it uses simple, native blood smears. These smears can be shipped via mail, reducing the impact of sample transport time on analysis, a critical factor for remote or pediatric patients requiring only minimal blood volumes [11,12,25].

This study evaluates the clinical utility of an automated IF-based diagnostic approach for IPDs. By standardizing the technique and integrating automated analysis, we aim to enhance workflow efficiency, reproducibility, and diagnostic accuracy using BSS and GT as disease models.

## 2. Materials and Methods

### 2.1. Collection and Preparation of Blood Samples for Analysis

Venous blood samples were collected into EDTA or sodium citrate tubes for various analyses, including DNA extraction, automated platelet counting, platelet aggregometry, flow cytometry, and blood smear preparation. This study was approved by the ethics committee of Bonn University of Medical Sciences (approval number 078/21). All patients provided written informed consent to participate in the study, which was conducted according to the Declaration of Helsinki.

To ensure high-quality peripheral blood smears, blood samples were drawn from a venipuncture site with minimal tourniquet application (<1 min). After collection, tubes were gently inverted multiple times to ensure thorough mixing with the anticoagulant, preventing clot formation [27]. All patient samples were processed directly for analysis. Samples from healthy controls were processed either directly or within four hours to ensure optimal preservation of cell morphology and platelet integrity. Blood smears were prepared using anticoagulated whole blood on glass slides with frosted edges (AA00000112E01MNZ10, Epredia, Leverkusen, Germany). Smears were air-dried for at least 20 min before further processing. May–Grünwald–Giemsa and IF staining were performed as previously described [25,26,28], with some modifications. Blood smears were fixed and permeabilized with 99.9% methanol at −30 °C for 1 min or acetone at −20 °C for 5 min. Slides were either processed immediately or stored at −20 °C until use.

**Figure 1:**
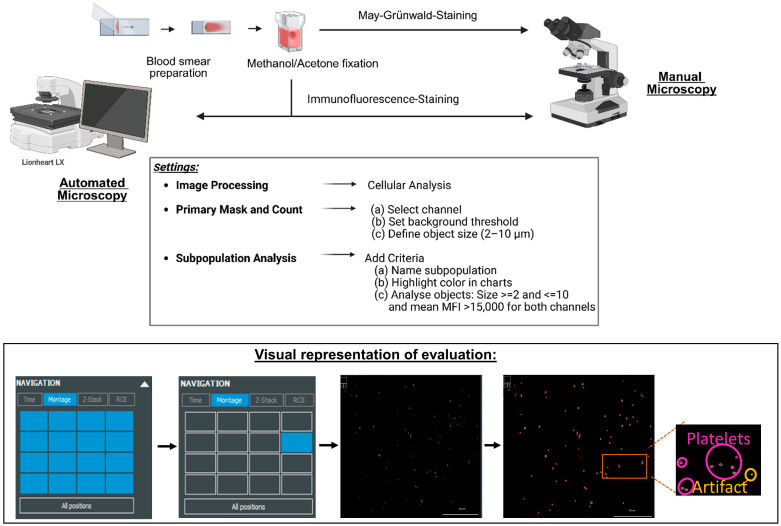
Workflow for automated immunofluorescence microscopy in the diagnosis of inherited platelet disorders.

The pre-marked smear sections were stained with directly labeled primary antibodies with a fluorescent dye or with appropriate secondary antibodies as detailed in Table S1. Double-fluorescence labeling targeting both the protein of interest and a platelet-specific marker was performed for each antibody combination. The binding specificity of the CD41 (P2) and CD42b antibodies to the surface of platelets was verified by manual microscopy (Figure S2). Blood smears were assessed by IF microscopy, and the expression of the protein of interest was assessed in comparison with a normal control stained in parallel and compared to the established cut-off (Figure 1).

While erythrocyte autofluorescence did not interfere with manual blood smear evaluation, it prevented an automated assessment of methanol-fixed smears due to high background noise. For methanol-fixed blood smears, erythrocyte autofluorescence was reduced using the Vector TrueVIEW Autofluorescence Quenching Kit (VEC-SP-8400, Biozol, Hamburg, Germany) via 2 min incubation according to the manufacturer’s instructions. Slides were mounted using VECTASHIELD Vibrance^®^ Antifade Mounting Medium containing DAPI (Figure S1). In contrast, acetone-fixed smears did not exhibit significant autofluorescence and could be analyzed without additional treatment.

### 2.2. Image Acquisition and Analysis

Microscopy was conducted using an automated imaging system (BioTek Lionheart LX, Agilent Technologies Inc., Waldbronn, Germany), and image acquisition was performed using the Gen 5 software (version 3.12.08, Agilent Technologies Inc.). Contrast and brightness were adjusted as needed to optimize the signal-to-noise ratio. To accurately identify platelet populations, thresholding and size gating were applied, and the processed data were exported for further statistical analysis. The automated analysis records 16 fields of view, evaluating between 100 and 500 platelets per sample. Fluorescence signals were acquired using the Lionheart LX automated imaging system. Channel spillover was minimized by appropriate filter selection and single-stain controls. Only double-positive events (green and red) were considered true platelet subpopulations expressing the antigen of interest (Figure 1).

Target antigen measurements were compared to control cut-off values, distinguishing normal from pathological platelets. Laboratory internal cut-offs were assessed based on the 2.5% quantile (0.025) as a reference point. Accordingly, 97.5% of the analyzed platelets from healthy donors show an MFI above the defined cut-off value. An unpaired *t*-test was used to compare means between two independent groups (e.g., control vs. patients). A *p*-value less than 0.05 was considered statistically significant. Quantitative measurements for fluorescence intensity and platelet size were exported; statistical analysis was conducted and figures were created using GraphPad Prism 10.2.2 software.

## 3. Results

### 3.1. Assessing Pre-Analytical Influences and Assay Variability on MFI Values

#### 3.1.1. Influence of Blood Smear Preparation Timing and Anticoagulant Selection on Result Accuracy

To assess the optimal time window for blood smear preparation and its impact on result variability, smears were prepared at multiple time points, up to 4 h after blood collection, from three healthy individuals (Controls 1–3). The target antibody CD41 P2, which targets the α-chain of CD41a (GPIIbα) within the intact GPIIb–IIIa complex, was selected. This choice allowed the evaluation of whether, as previously reported [26], the stability of this integrin complex differs between citrate- and EDTA-anticoagulated blood.

**Figure 2:**
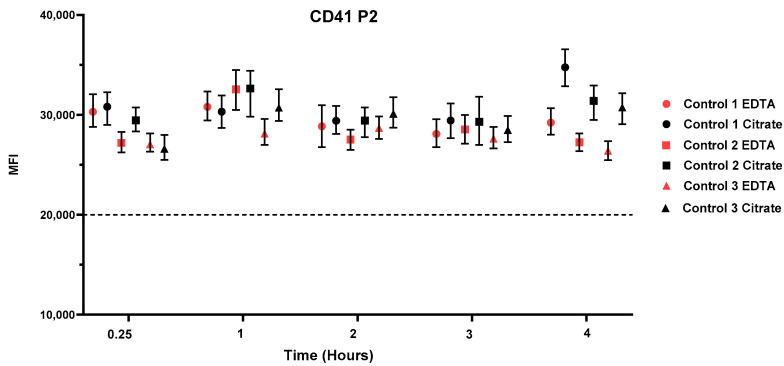
Stability of the integrin αIIbβ3 complex (GPIIb–IIIa) in EDTA or citrate anti-coagulated blood. The graph shows the median green fluorescence intensity of CD41P2-labeled platelets over a blood smear preparation period of up to four hours following blood collection. Blood smears were prepared from anticoagulated blood using either EDTA (red symbols) or citrate (black symbols). Control 1 is represented by circles, Control 2 by squares, and Control 3 by triangles. The dashed horizontal line indicates the antibody cut-off threshold.

The results are shown in Figure 2. The analysis revealed that blood smear analysis remains reliable within the first 3 h of collection. Up to 3 h after blood collection, integrin stability remained consistent across both anticoagulants, with no statistically significant difference observed, staying within the ±25% experimental variability range. However, at 4 h, all three individuals showed higher MFI values in citrate-anticoagulated blood samples, while EDTA-anticoagulated blood smears exhibited no change in MFI values compared to earlier assessments. Beyond the 4 h time window, the blood smears were no longer analyzable. These findings highlight the importance of timely processing for accurate results and confirm that the choice of anticoagulant has no significant impact on smear quality (Figure 2).

#### 3.1.2. Inter-Assay Evaluation

**Figure 3:**
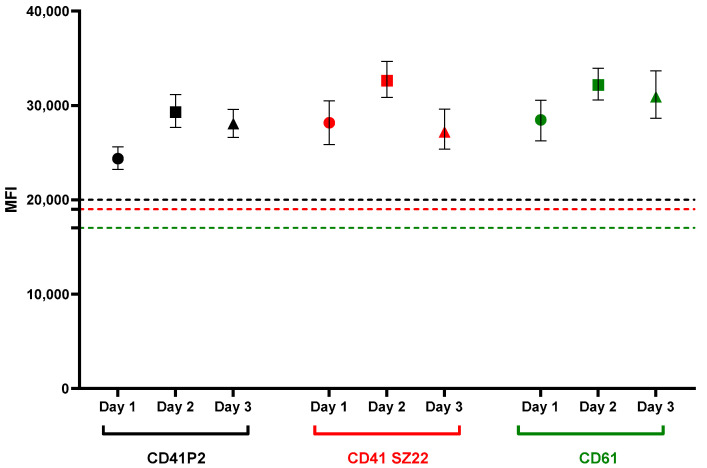
Comparison of inter-assay signal intensity variations in the integrin αIIbβ3 complex (GPIIb–IIIa). The graph illustrates the mean green fluorescence intensity of platelets stained with CD41P2 (black), CD41 SZ22 (red), and CD61 (green) on blood smears prepared from a healthy individual on three different days. Symbols represent different biological replicates: circles for day 1, squares for day 2, and triangles for day 3. The dashed horizontal lines indicate the respective antibody cut-off thresholds. Error bars represent the standard deviation (SD).

Next, we calculated the inter-assay coefficient of variation (CV). Blood smears from a healthy individual (Control 4) were analyzed on three different days, and the mean values from approximately 250 platelets were plotted. To strengthen the analysis, two additional antibodies were included (Figure 3).

The results showed CVs of 9.8%, 9.4%, and 6% for CD41 P2, CD41 SZ22, and CD61 antibodies, respectively (Table 1).

### 3.2. Establishing the Parameters for Automated Assessment of the Platelets

**Figure 4:**
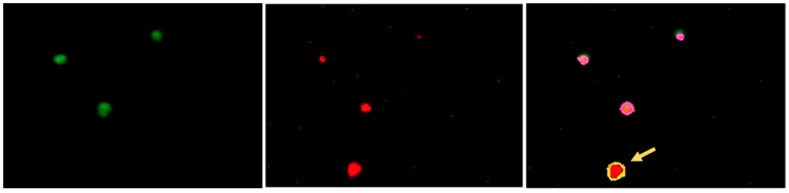
Automated subpopulation analysis using automated microscopy on Lionheart LX. This figure presents an automated analysis of platelet subpopulations through dual-IF labeling. The left image displays green-positive platelets (CD61), the middle image shows red-stained cells (CD63), and the rightmost image is an overlay combining both fluorescence channels. In the overlay, pink circles indicate CD61⁺/CD63⁺ double-positive platelets automatically segmented and counted by the analysis Gen 5 software (version 3.12.08, while yellow circles (arrow) highlight artifacts lacking double fluorescence, which are excluded from the analysis.

Next, we established a quantitative rapid image-based protocol for automated subpopulation analysis of platelets on IF-stained blood smears. Predefined analysis protocols allowed the microscope to scan slides at fixed positions and capture images. Data evaluation followed a rule-based approach, analyzing fluorescence signals within a predefined platelet size range (2–10 µm) and threshold (>15,000 MFI). First, all platelets were identified using a detection marker, followed by the inclusion of fluorescence signals from a secondary antibody. This approach minimized background artifacts and enabled the identification of platelets with absent or reduced target antigen expression. The automated analysis and antibodies were validated using patient samples. Since no established reference cut-off values were previously available, an initial reference group of healthy donors was assessed to define internal laboratory thresholds. Fluorescence signal evaluation was conducted by comparing results with a control sample from a healthy donor and referencing internal laboratory cut-offs (Figure 4).

### 3.3. Quantitative Image-Based Confirmation of BSS and GT Diagnosis on IF-Stained Blood Smears

To validate the image-based diagnostic approach, we analyzed patients with a definitive genetically confirmed diagnosis of BSS and GT by evaluating all glycoproteins involved in the assembly of the GPIb–IX–V and GPIIb–IIIa complex, respectively.

#### 3.3.1. Validation of the Automated IF Approach for the Diagnosis of BSS

For this purpose, two patients (IP1 and IP2) with either homozygous or compound heterozygous missense variants were enrolled. The characteristics of the patients are summarized in Table 2.

For both patients, the quantitative approach was performed for three glycoproteins responsible for the (GP) Ib–IX–V complex involved in the assembly of the GPIbα (CD42b), GPIbb (CD42c), and GPIX (CD42a) subunits. For all antibodies, the healthy control showed fluorescence intensities above the cut-off, indicating the normal expression of the GPIb–IX–V complex.

Patient IP1, carrying a homozygous p.Cys24Arg variant in *GP9,* showed almost no CD42a signal, indicating the absence of GPIX expression. The expression of this subunit was reduced but still detectable in patient IP2, with the compound heterozygous missense variants (p.Cys24Arg, p.Asn61Ser) in *GP9* showing reduced but detectable CD42a expression.

**Figure 5:**
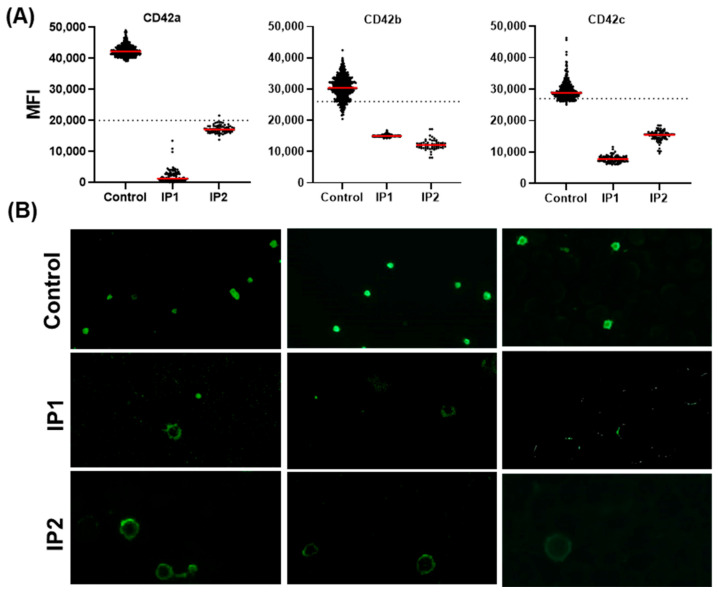
Validation of Bernard–Soulier syndrome via fluorescence intensity detection of the GPIb–IX–V complex. (**A**) The scatter plots (top row) show the mean green fluorescence intensity for the GPIX (CD42a), GPIbα (CD42b) and GPIbb (CD42c) subunit markers in the control group and in two patients. Patient IP1 and patient IP2 are compared to the control sample. The dashed horizontal lines indicate the respective antibody cut-off thresholds. In the red channel, CD63 was used as the second platelet marker. (**B**) The bottom panels display fluorescence microscopy images of the platelet staining.

Both patients showed markedly reduced fluorescence levels below the cut-off threshold, suggesting a significant reduction in or absence of CD42b and CD42c expression. Overall, the results confirm a strong reduction in CD42a, CD42b, and CD42c expression in both patients compared to the control group (Figure 5A). The documented images of the automated microscopy of the IF-stained blood smears were in line with the MFI values obtained for all three subunits (Figure 5B). The reduced expression of the surface marker CD42b in flow cytometry (Table 2) matched the results obtained from the IF-stained blood smears, demonstrating strong concordance between flow cytometry and IF analysis.

#### 3.3.2. Validation-Automated IF Approach for Diagnosis of GT

Two GT IPs with different genetic constellations were enrolled: the first patient (IP4) had a homozygous missense variant (p.Leu118His), whereas the second patient (IP5) harbored compound heterozygous missense (p.Asp314Ala) and nonsense (p.Gln40Ter) variants in the ITGB3 gene. The characteristics of the patients are summarized in Table 2.

For all patients, the quantitative approach was performed for the integrin αIIbβ3 complex GPIIb–IIIa (CD41 P2), the assembly subunits, the glycoproteins GPIIb (CD41 SZ22) and GPIIIa (CD61). For all antibodies, the healthy control shows fluorescence intensities above the cut-off, indicating the normal expression of the GPIIb–IIIa complex.

**Figure 6:**
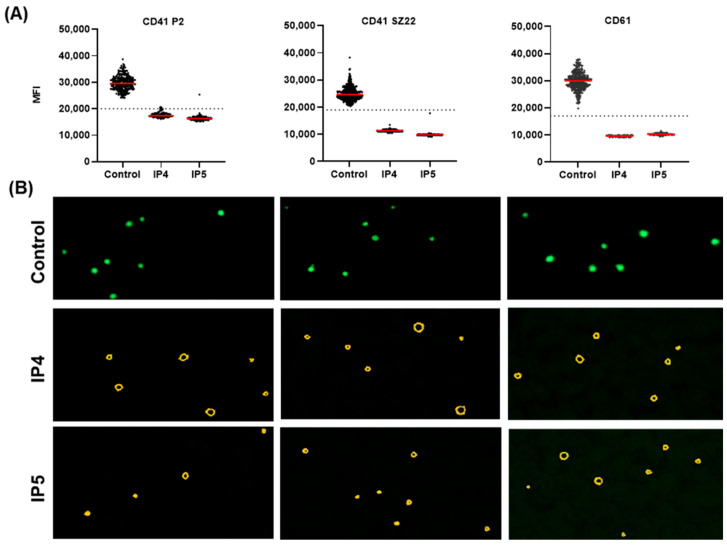
Validation of Glanzmann thrombasthenia via fluorescence intensity detection of the integrin αIIbβ3 complex (GPIIb–IIIa). (**A**) The scatter plots (top row) show the mean green fluorescence intensity for CD41 P2 detecting the GPIIb–III complex and the assembly subunits CD41 SZ22 (GPIIb) and CD61 (GPIIIa). The dashed horizontal lines indicate the respective antibody cut-off thresholds. (**B**) The bottom panels display fluorescence microscopy images of platelet staining. The green channel shows the indicated antibodies for the integrin αIIbβ3 complex, and in the red channel, CD63 was used as the second platelet marker (data shown in Supplementary Materials, Figure S3).

For both IPs, the almost absent fluorescence below the cut-off threshold for all three antibodies indicates the complete loss of the GPIIb–IIIa complex, consistent with a GT phenotype characterized by absent integrin αIIbβ3 surface expression. The documented images of the automated microscopy of the IF-stained blood smears were in line with the MFI values obtained for both subunits and the GPIIb–IIIa complex (Figure 6A,B). The abundant expression of the surface marker CD41 (clone SZ22) and the GPIIb–IIIa complex (P2 clone) in flow cytometry (Table 2) corresponds with the findings from the IF-stained blood smears, indicating a strong agreement between flow cytometry and IF analysis.

#### 3.3.3. Evaluating Quantitative Defects of BSS and GT Using the Established Automated IF Approach

**Figure 7:**
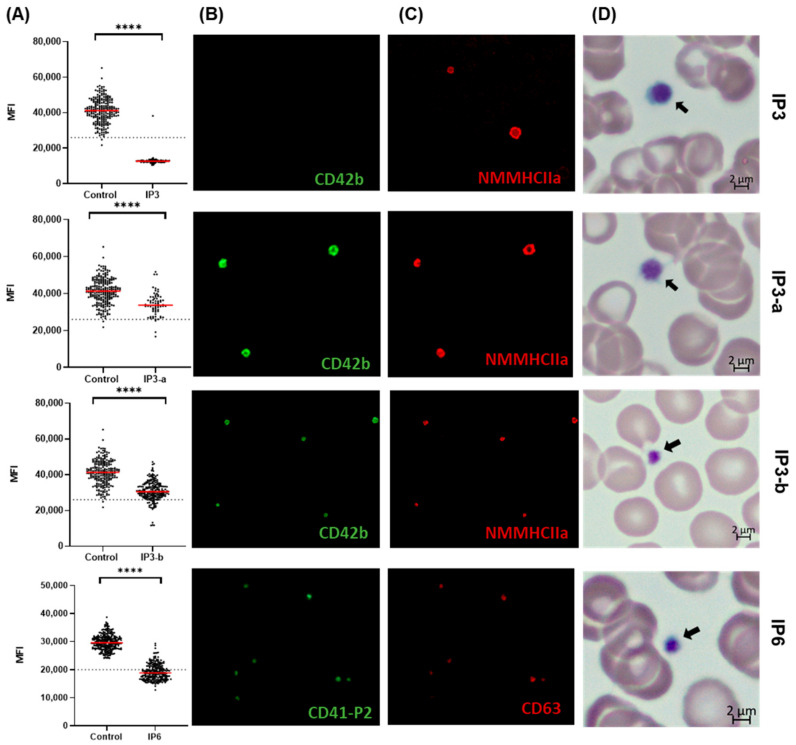
Evaluating the expression of GPIbα (CD42b) and GPIIb/IIIa (CD41 P2) in a Bernard–Soulier syndrome family and Glanzmann thrombasthenia type II patient. (**A**) The scatter plots (left column) show the mean green fluorescence intensity for either CD42b or CD41 P2. The dashed horizontal lines indicate the respective antibody cut-off thresholds. Asterisks denote statistically significant differences compared to the control (unpaired *t*-test): *p* < 0.0001 (****). (**B**,**C**) The middle panels display fluorescence microscopy images of platelet staining with CD42b or CD41 P2 in the green channel and CD63 or NMMHCIIa (Myosin) in the red channel. (**D**) The right panel shows the respective light microscopy features of the platelets. The arrow indicates the platelets.

To further evaluate the established automated IF protocol, two extra IPs with BSS (IP3) and GT (IP6) were evaluated. IP3 harbored compound heterozygous variants in GP9. For this patient, blood smears of the parents (IP3-a, mother of IP3, and IP3-b, father of IP3) were evaluated to assess the utility of the test for heterozygous defects. The characteristics of the patients are summarized in Table 2. The analysis revealed that the BSS patient exhibited a complete loss of expression of the GPIbα subunit (Figure 7A). On the other hand, the heterozygous parents had a profound reduction in CD42b expression compared to the healthy controls (*p* < 0.0001). Quantitative analysis showed a highly significant decrease in mean GFP fluorescence intensity in heterozygous carriers of the genetic defect (Figure 7B,C). Hence, differences in platelet size and numbers were observed depending on the detected variant. While IP3-a showed enlarged platelets and a reduced number of platelets (from borderline to normal-range both on the blood smears and the blood analyzer), IP3-b showed no morphological abnormalities and normal MPV values (Figure 7D).

The next analyzed patient (IP6) with a compound heterozygous defet in the ITGA2B gene (nonsense variant p.Tyr319Ter and an in-frame duplication, p.Glu776_Ala777dup) was diagnosed with GT type II and demonstrated a marked reduction in CD41 P2 expression, indicative of impaired GPIIb–IIIa complex formation (*p* < 0.0001), but not a complete absence of the αIIbβ3 integrin complex, which aligns with a diagnosis of GT type II. Collectively, these data confirm the expected surface glycoprotein deficiencies in patients with BSS and GT, confirmed using automated IF analysis.

## 4. Discussion

In this study, we aimed to validate an automated IF-based image analysis method for the diagnosis of IPDs, with a particular focus on BSS and GT. By combining quantitative fluorescence microscopy with targeted glycoprotein antibody staining, we show that this approach reliably identifies characteristic expression patterns associated with both disorders.

The diagnostic performance of the method appears promising, demonstrating strong sensitivity and specificity across the tested samples. Notably, even in cases with reduced but not completely absent expression, such as GT type II or heterozygous BSS, the assay was able to capture significant differences in fluorescence intensity, supporting its use for fine phenotypic resolution. Importantly, inter-assay variability was low, with coefficients of variation well below 10%, confirming the reproducibility of the protocol.

In our pre-analytical evaluation, we quantitatively demonstrated that the choice of anticoagulant—either EDTA or citrate [29]—had no significant impact on the stability or detection of the tested integrin αIIbβ3 complex (GPIIb–IIIa) within the first three hours post-collection. While the results were still within acceptable limits, this finding shows the importance of recording the exact time of processing, especially in settings when different operators uptake and prepare the blood smears. Using CD41 P2 as a representative antibody targeting the α-chain of GPIIb, MFI values remained within the acceptable variability range (±25%) across both anticoagulants. These findings confirm that either anticoagulant can be used without compromising the assay’s diagnostic reliability, providing flexibility in clinical sample handling and transportation logistics, in contrast to flow cytometry, where only citrated blood can be used [26]. This is particularly relevant for centers without specialized facilities, as it expands the range of acceptable pre-analytical conditions while maintaining analytical accuracy. Inter-laboratory cooperation between specialized centers could help standardize immunofluorescence microscopy, establishing higher quality control standards [30].

Another concern with immunofluorescence on blood smears is the issue of erythrocyte autofluorescence, which can obscure or confound signal interpretation. While this is a valid consideration, we were able to minimize its impact by using a quencher agent. Also, the dual-channel fluorescence approach combined with automated subpopulation analysis allowed for a descriptive evaluation of the data. Our algorithm identifies platelet-sized structures and includes only those with sufficient signal intensity in both the target and reference channels, effectively excluding background fluorescence signals from erythrocytes and debris. This rule-based gating strategy, along with threshold-based filtering, allows for the robust and reproducible identification of platelets, ensuring that the measured fluorescence signal truly reflects target glycoprotein expression. While the current automation reliably detects platelet disorders involving antigen expression defects, structural platelet abnormalities still require manual evaluation. Future artificial intelligence (AI)-driven image recognition could help fully automate this process.

Nonetheless, several advantages make this method promising for broader clinical application. The technique requires only a minimal volume of blood (<100 µL), making it particularly suitable for neonates or patients with difficult venous access [25,26]. Its minimal sample requirement also supports use in family studies, especially where genetic testing may be ethically or technically challenging. In such cases, this method can serve as a preliminary screening step to guide or prioritize downstream genetic investigations, especially for evaluating the pathogenicity of variants of uncertain significance (VUS) [25].

A major strength of this approach is its accessibility and scalability. Since only blood smears are required, samples can be shipped over long distances from peripheral centers to specialized laboratories, facilitating access to second-level platelet diagnostics even in regions without dedicated on-site facilities. The flexibility to include multiple antibodies—targeting entire glycoprotein complexes—enhances diagnostic precision compared to conventional flow cytometry, which often limits analysis to a few selected markers [31]. This is particularly important for comprehensive glycoprotein profiling and has potential applications in contexts such as human platelet antigen (HPA) compatibility and alloimmunization risk assessment [32]. By detecting reduced or absent glycoprotein expression, this assay could help identify at-risk individuals, improve donor matching, and guide preventive strategies in transfusion and pregnancy care. Moreover, in direct comparison with flow cytometry, IF staining requires significantly lower antibody concentrations, making this method a more cost-effective alternative. Furthermore, IF microscopy allows for the integration of both quantitative analysis and morphological platelet evaluation in a single assay.

With standardization, this approach could become part of a broader diagnostic workflow, serving as a rapid and scalable intermediate step between routine hematological screening and genetic confirmation. Especially in settings where molecular testing is delayed or unavailable, IF microscopy can guide early clinical suspicion and targeted genetic investigations.

Despite its strengths, this study has several limitations. The most prominent is the low number of patient samples (n = 8). Given the rarity of these disorders, we opted to include only well-characterized cases from our center with complete clinical, phenotypic, and genetic data. While this ensured internal validity, it underscores the need for future multicenter collaborations to expand the dataset and strengthen external generalizability. Further data collection is needed to refine specificity, sensitivity, and reference intervals. Furthermore, this method provides no information on in vivo platelet functionality. Pre-analytical artifacts, such as platelet activation during blood draw or smear preparation, can also impact signal interpretation. In this regard, flow cytometry offers a diagnostic advantage, as platelet activation can be detected using the CD62P activation marker [33]. Therefore, IF is a complementary screening method and should be supplemented with flow cytometric analysis in cases of abnormal findings [34]. While our pre-analytical analysis showed stable integrin expression up to 3 h post-collection, delayed smear preparation or suboptimal handling may still introduce variability. Moreover, the assay remains operator-dependent, emphasizing the need for proper training and standard operating procedures. To achieve more standardized and higher-quality blood smears, the use of automated smearing systems offers a promising solution for improving reproducibility [35]. The establishment of proficiency-testing programs and inter-laboratory quality control remains an unmet need [11].

## 5. Conclusions

This study demonstrates that immunofluorescence microscopy, when combined with standardized protocols and automated analysis, is a valuable diagnostic tool for inherited platelet disorders. Its application enabled accurate identification not only of classical BSS and GT cases, but also of milder phenotypes such as GT type II and heterozygous BSS. The future integration of AI-based image analysis could further enhance diagnostic efficiency, objectivity, and diagnostic accuracy, making this approach especially useful in settings where traditional diagnostic methods are limited.

## Figures and Tables

**Table 1:** Inter-assay coefficient of variation (CV) from a healthy individual analyzed on three different days.

Antibody	Day 1	Day 2	Day 3	Inter-Assay CV		
	MFI ^1^			Mean MFI	St DV ^2^	Inter CV%
CD41 P2	24,431	29,485	28,577	27,498	2694	9.8
CD41 SZ22	28,461	32,885	27,690	29,679	2803	9.4
CD61	28,516	32,118	30,969	30,534	1840	6

^1^ MFI: Mean fluorescence intensity, ^2^ St DV: standard deviation.

**Table 2:** Characteristics and laboratory parameters of the index patients (IPs). Normal ranges are placed in curly brackets {}.

IPD		Bernard–Soulier Syndrome					Glanzmann Thrombasthenia		
Patients		IP1	IP2	IP3	IP3-a	IP3-b	IP4	IP5	IP6
Platelet count {150–370} × 10^3^/µL		78	3	48	153	224	207	291	306
MPV {8.5–11.5} fL		-	-	-	13.5	10.9	12.3	11.2	10.0
Genetic defect	Gene	*GP9*	*GP9*	*GP9*	*GP9*	*GP9*	*ITGB3*	*ITGB3*	*ITGA2B*
	HGVS c.	c.[70T>C]; [70T>C]	c.[70T>C];[ 182A>G]	c.[70T>G]; [182A>G]	c.[182A>G];[=]	c.[70T>G];[=]	c.[353T>A]; [353T>A]	c.[118C>T]; [941A>C]	c.[957T>A]; 2326_2331dupGAGGCC
	HGVS p.	p.(Cys24Arg)	p.(Cys24Arg; Asn61Ser)	p.(Cys24Arg; Asn61Ser)	p.(Asn61Ser)	p.(Cys24Arg)	p.(Leu118His)	p.(Gln40Ter; Asp314Ala)	p.(Y319Ter; p.Glu776_Ala777dup)
	Zygosity	homozygous	compound heterozygous	compound heterozygous	heterozygous	heterozygous	homozygous	compound heterozygous	compound heterozygous
	Variant	missense	missense	missense	missense	missense	missense	nonsense, missense	nonsense, in-frame duplication
Flow Cytometry	CD41 P2	N	N	N	N	n.d.	P	P	P
	CD41 SZ22	N	N	N	N	n.d.	P	P	P
	CD42b	P	P	P	P	n.d.	N	N	N
Automated IF Microscopy	CD41 P2	N	N	n.d.	n.d.	n.d.	P	P	P
	CD41 SZ22	N	N	n.d.	n.d.	n.d.	P	P	n.d.
	CD61	N	N	n.d.	n.d.	n.d.	P	P	n.d.
	CD42a	P	P	n.d.	n.d.	n.d.	N	N	n.d.
	CD42b	P	P	P	P	P	N	N	n.d.
	CD42c	P	P	n.d.	n.d.	n.d.	N	N	n.d.

IF: Immunofluorescence; MPV: mean platelet volume; fL: femtoliter; IPD: inherited platelet disorders; n.d. not done; P: Pathologic; N: Normal.

## Data Availability

The dataset is available on request from the authors.

## Supplementary Materials

The following supporting information can be downloaded at https://www.mdpi.com/article/10.3390/genes16060621/s1: Figure S1: Quenching the autofluorescence of erythrocytes for automated analysis of the methanol-fixed blood smears using CD41 marker. Figure S2: Surface localization for CD41 and CD42b staining on blood smears from healthy donors. Figure S3: Fluorescence microscopy analysis of platelet integrin subunits in patients IP4 and IP5. Table S1: Overview of primary and secondary antibodies used for immunofluorescence staining of platelet surface markers.

## Abbreviations

The following abbreviations are used in this manuscript:

AI	Artificial intelligence
ADP	Adenosine diphosphate
BSS	Bernard–Soulier Syndrome
CD	Cluster of differentiation (used to identify cell surface molecules, e.g., CD41, CD42a)
CV	Coefficient of variation
DAPI	4′,6-diamidino-2-phenylindole (DNA fluorescent stain)
DNA	Deoxyribonucleic acid
EDTA	Ethylenediaminetetraacetic acid (anticoagulant)
FACS	Fluorescence-activated cell sorting (flow cytometry technique)
*GP1BA*	Gene encoding GPIbα
*GP1BB*	Gene encoding GPIbb
*GP9*	Gene encoding GPIX
GPIb/IX/V	Glycoprotein Ib–IX–V Complex
GPIbb	Glycoprotein Ib beta
GPIbα	Glycoprotein Ib alpha
GPIIb/IIIa	Glycoprotein IIb–IIIa complex (αIIbβ3 integrin)
GPIX	Glycoprotein IX
GT	Glanzmann thrombasthenia
HGVS	Human Genome Variation Society
HLA	Human leukocyte antigen
HPA	Human platelet antigen
IF	Immunofluorescence
IPDs	Inherited platelet disorders
IPF	Immature platelet fraction
iPFDs	Inherited platelet function disorders
*ITGA2B*	Integrin subunit alpha 2b (gene encoding GPIIb)
*ITGB3*	Integrin subunit beta 3 (gene encoding GPIIIa)
MFI	Mean fluorescence intensity
MPV	Mean platelet volume
SD	Standard deviation
VUS	Variant of unknown significance
