# Supplementary material for: Automated Quantitative Immunofluorescence Microscopy Approach for Diagnosis of Hereditary Thrombopathies: A Proof of Concept Using Bernard–Soulier Syndrome and Glanzmann Thrombasthenia
Source: Genes (Basel). 2025 May 23;16(6):621. doi: 10.3390/genes16060621 (PMC12192073; doi:10.3390/genes16060621)
Supplement: Supplementary file 1 [file genes-16-00621-s001.zip › genes-3618243-supplementary.pdf]

Supplementary Materials

Table S1. Overview of primary and secondary antibodies used for immunofluorescence staining of platelet surface markers.

	Antibody	Clone	Isotype /Species	Conjugate	Company	Catalog Number
GPIIb/IIIa Complex	CD41	P2	IgG_1 Mouse	FITC	Beckman Coulter	IM0649U
	CD41	SZ22	IgG_1 Mouse	FITC	Beckman Coulter	IM1756U
	CD61	Y2/51	IgG_1 Mouse	FITC	Thermo Fisher	MA5-16684
GPIb-IX-V Complex	CD42a	FMC-25	IgG_1 Mouse	-	Santa Cruz	sc-59052
	CD42b	SZ2	IgG_1 Mouse	-	Enzo Life Sciences Inc.	ALX-805-026-C100
	CD42c	Gi27	IgG_{2b} Mouse	-	Beckman Coulter	IM0649U
LAMP3	CD63	H5C6	IgG_1 Mouse	Alexa Fluor™ 594	BioLegend	353034
NMMHCIIa	NMMHCIIa	Poly19099	IgG Rabbit	-	BioLegend	909901
Secondary Antibodies	Anti-Mouse	Polyclonal	IgG Goat	FITC	Invitrogen	F2761
Secondary Antibodies	Anti-Rabbit	Polyclonal	IgG Goat	Alexa Fluor™ 594	Invitrogen	A11012

 https://doi.org/10.3390/xxxxx

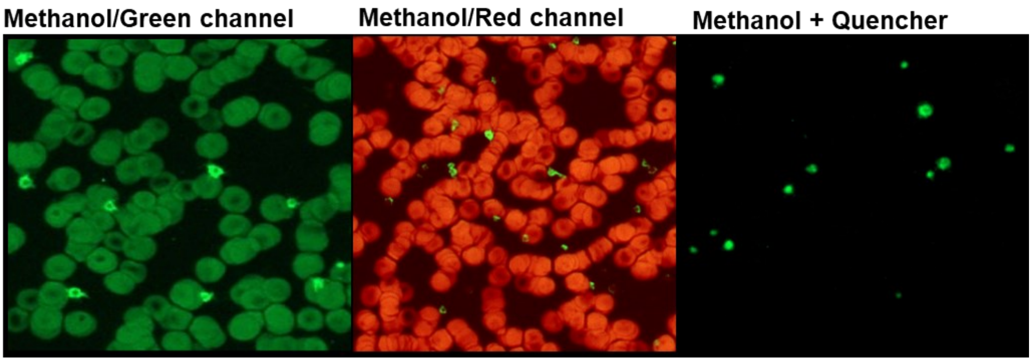

Figure S1: Quenching the autofluorescence of erythrocytes for automated analysis of the methanol-fixed blood smears using CD41 marker. This figure illustrates the background noise signal due to the auto fluorescence of the erythrocytes of the methanol-based fixed samples. Methanol fixation preserves CD41 signal in both green and red channels but also shows considerable background. In contrast, methanol fixation with a quencher significantly reduces background fluorescence while maintaining platelet-specific signals.

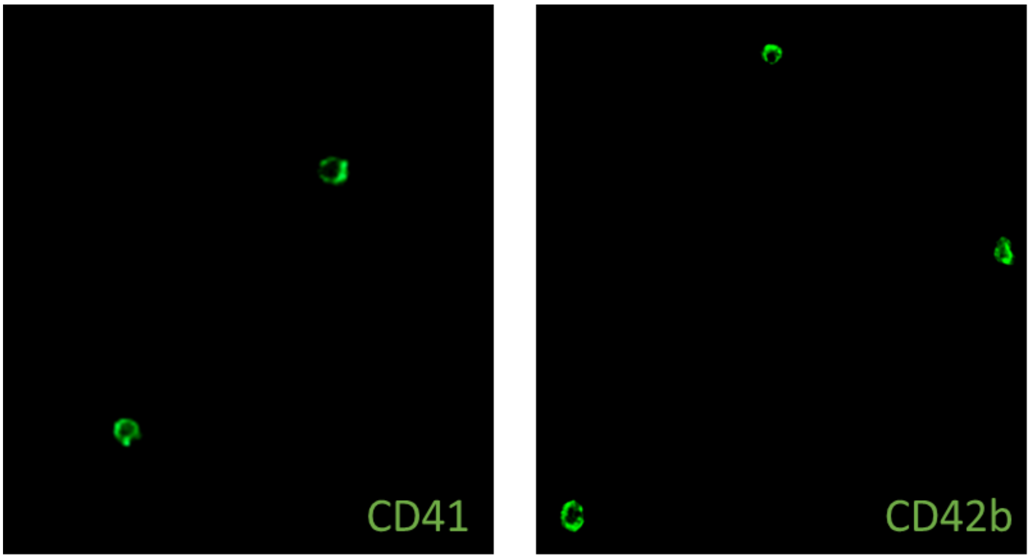

Figure S2. Surface Localization for CD41 and CD42b Staining on Blood Smears from Healthy Donors. Representative fluorescence images showing the surface expression of CD41 (left) and CD42b (right) on platelets in peripheral blood smears from healthy control donors. Blood smears were fixed and stained using fluorophore-conjugated antibodies. The distinct ring-shaped staining pattern confirms that both CD41 and CD42b are localized to the platelet surface, with no detectable intracellular signal.

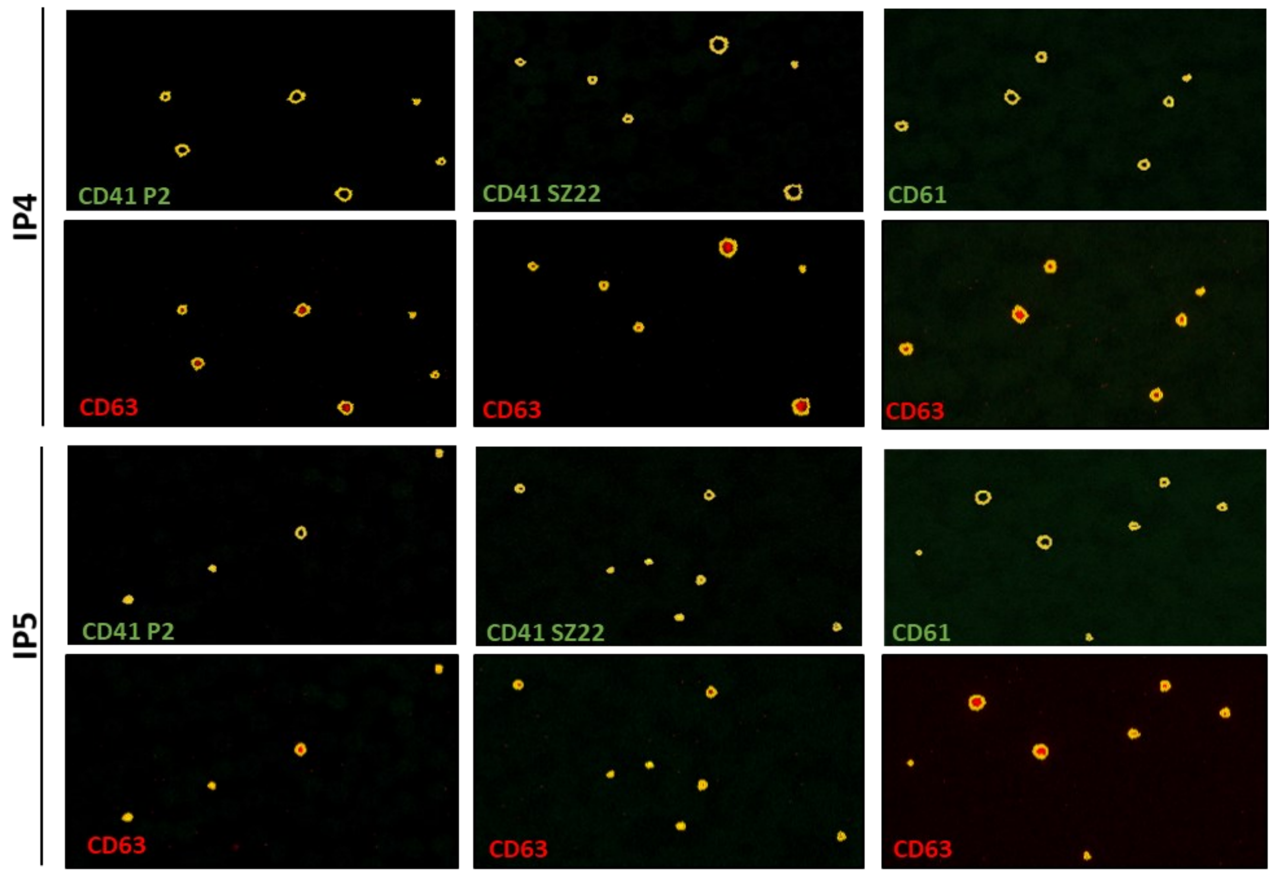

Figure S3: Fluorescence microscopy analysis of platelet integrin subunits in patients IP4 and IP5. Representative images display immunofluorescent staining of platelets from patients IP4 (top panels) and IP5 (bottom panels). Antibodies against integrin subunits CD41 P2, CD41 SZ22, and CD61 are shown in green, while CD63 is shown in red. Fluorescence signals were acquired using the Lionheart LX automated imaging system. Yellow circular outlines highlight platelets and are generated by the imaging software based on CD63 signal.
